# Ketone body β-hydroxybutyrate (BHB) preserves mitochondrial bioenergetics

**DOI:** 10.1038/s41598-023-46776-8

**Published:** 2023-11-11

**Authors:** I. Llorente-Folch, H. Düssmann, O. Watters, N. M. C. Connolly, Jochen H. M. Prehn

**Affiliations:** 1 Department of Physiology and Medical Physics, Royal College of Surgeons in Ireland, 123 St. Stephen’s Green, Dublin 2, Ireland; 2 Centre for Systems Medicine, Royal College of Surgeons in Ireland, Dublin 2, Ireland; 3 Department of Basic Sciences of Health, Area of Biochemistry and Molecular Biology, Universidad Rey Juan Carlos, 28922 Alcorcón, Madrid Spain; 4 SFI FUTURE-NEURO Research Centre, Royal College of Surgeons in Ireland, Dublin 2, Ireland

**Keywords:** Neuroscience, Physiology

## Abstract

The ketogenic diet is an emerging therapeutic approach for refractory epilepsy, as well as certain rare and neurodegenerative disorders. The main ketone body, β-hydroxybutyrate (BHB), is the primary energy substrate endogenously produced in a ketogenic diet, however, mechanisms of its therapeutic actions remain unknown. Here, we studied the effects of BHB on mitochondrial energetics, both in non-stimulated conditions and during glutamate-mediated hyperexcitation. We found that glutamate-induced hyperexcitation stimulated mitochondrial respiration in cultured cortical neurons, and that this response was greater in cultures supplemented with BHB than with glucose. BHB enabled a stronger and more sustained maximal uncoupled respiration, indicating that BHB enables neurons to respond more efficiently to increased energy demands such as induced during hyperexcitation. We found that cytosolic Ca^2+^ was required for BHB-mediated enhancement of mitochondrial function, and that this enhancement was independent of the mitochondrial glutamate-aspartate carrier, Aralar/AGC1. Our results suggest that BHB exerts its protective effects against hyperexcitation by enhancing mitochondrial function through a Ca^2+^-dependent, but Aralar/AGC1-independent stimulation of mitochondrial respiration.

## Introduction

Up to 65% of individuals with epilepsy will have their seizures controlled with antiepileptic drugs or enter spontaneous remission during their lifetime^1^. However, this leaves a large percentage of patients who are refractory to drug therapy^2^. Ketogenic diet (KD) has been shown to be beneficial for the treatment of refractory epilepsy^3–6^. This therapeutic approach has also been proposed for the treatment of other neurological diseases such as Alzheimer’s disease^7^, amyotrophic lateral sclerosis^8^, Huntington’s disease^9^, autism^10^, Parkinson’s disease^11^ and ARALAR/AGC1 deficiency^12^. Many clinical studies have confirmed the beneficial effect of a KD, however, the mechanisms^13^ through which a KD confers its seizure-reducing, protective effects remain unknown.

KD has a high fat content (80–90%), with little but sufficient protein and a drastic reduction in carbohydrate content. The KD is mainly composed of triglycerides. Long-chain fatty acids (LCFA) and medium-chain fatty acids (MCFA), which are metabolised mainly in the liver, give rise to ketone bodies (acetone, acetoacetate and 3-beta-hydroxybutyrate). 3-beta-hydroxybutyrate (BHB) is the most abundant ketone body in mammals^14^. Emerging evidence shows that BHB is not only a passive carrier of energy but also has a variety of signalling functions, both at an intracellular and cell surface level, that can affect gene expression, lipid metabolism, neuronal function, and metabolic rate^14^.

A prime function of mitochondria is to reduce oxygen to water during oxidative phosphorylation (OXPHOS) in order to produce energy. In excitable cells like neurons, calcium (Ca^2+^) regulates this process^15–17^. ARALAR/AGC1, the regulatory component of the malate–aspartate NADH shuttle (MAS), operates in neurons^18^, is activated by cytosolic Ca^2^^+^ within the nanomolar range^18–21^ and is essential for OXPHOS regulation by Ca^2+^^22–24^. The aim of this study was to investigate the mechanisms underlying the bioenergetic effects of BHB in neurons cultured in physiological oxygen conditions (5%), and to explore the protective effects of BHB during glutamate-induced Ca^2+^ signalling, and its dependence on Aralar/AGC1 activity.

## Material and methods

### Materials and reagents

Fetal bovine serum (FBS), horse serum (HS), B27 supplement, minimal essential medium (MEM), Neurobasal medium, Neurobasal medium-A, Opti-MEM medium, Lipofectamine 3000, tetramethylrhodamine methyl ester (TMRM), Fluo-4 AM, and 1,2-*bis*-(o-aminophenoxy)-ethane-*N*,*N*,*N*′,*N*′-tetraacetic acid, tetraacetoxymethyl ester (BAPTA-AM) were from Invitrogen (Bio Sciences). D-glucose, (±)-Sodium 3-hydroxybutyrate (BHB)**,** L-Glutamic acid monosodium salt monohydrate (Glu), poly-D-lysine hydrobromide and cytosine arabinofuranoside (Ara-C) were from Sigma-Aldrich (Dublin, Ireland). Aminooxy acetic acid, hemihydrochloride (AOAA) were from Calbiochem. Seahorse XFe96 sensor cartridges, microplates, calibrant and DMEM medium were from Agilent. Bioenergetics reagents: oligomycin (Oli), Carbonyl cyanide-4-(trifluoromethoxy)phenylhydrazone (FCCP), antimycin (A) and rotenone (R) were from Sigma-Aldrich (Dublin, Ireland).

### Neuronal cell culture

Primary cultures of cortical or hippocampal neurons were prepared at embryonic day 16–18, as described previously^25^. First, the pregnant female C57BL/6 J wild‐type mice, embryonic gestation day 16–19 (E16-E18) were sacrificed by cervical dislocation and a hysterectomy of the uterus was performed. Rapid decapitation of the 5–6 embryos was carried out and the cerebral cortices and/or hippocampi were isolated and pooled in a dissection buffer on ice (1 × PBS with 0.25% glucose and 0.3% BSA). All animal work was performed with ethics approval (REC202002001) and under licenses granted by the Health Products Regulatory Authority in accordance with European Communities Council Directive (86/609/EEC), and procedures were reviewed and approved by the RCSI Research Ethics Committee. For the data presented in this study a total of 24 animals were required. All methods were carried out in accordance with relevant guidelines and regulations. The study is reported in accordance with ARRIVE guidelines. Then the tissue was incubated with 0.25% trypsin–EDTA at 37 °C for 15 min. After the incubation period, the trypsinisation was ceased by the addition of plating media (PM). The PM is comprised of MEM with 5% FBS, 5% HS, 100 U mL^−1^ penicillin/streptomycin, 0.5 mM L-glutamine and 0.45% (w/v) D-glucose. The neurons were gently dissociated using a Pasteur pipette and after centrifugation at 1500 rpm for 3 min, the medium containing trypsin was aspirated. The remaining cell pellet was re-suspended in fresh PM, seeded on poly-D-lysine-coated plates (final concentration of 50 μg/mL or 10 μg/mL on glass or plastic surface, respectively) and then incubated at 37 °C and 5% CO_2_ in ambient O_2_. Plating neuronal density varied from 1.2 to 1.5 × 10^6^ cells per sterile WillCo dish (WillCo Wells B.V.) of 35 mm diameter and 5.0 × 10^4^ cells/well in Seahorse XF96 well plates. After 24 h, PM was exchanged with 50% feeding medium (Neurobasal containing 100 U mL^−1^ penicillin/streptomycin, 2% B27, and 2 mM L-glutamine) and 50% fresh PM, with addition of the mitotic inhibitor, cytosine arabinofuranoside (Ara-C) (480–500 nM). At this point, cells used to study the effect of physiological O_2_ concentration were transferred to a BioSpherix Xvivo × 3 hypoxia chamber at 37 °C and 5% CO_2_ in 5% O_2_, while matched cell cultures were placed in a standard incubator at 37ºC, 5% CO_2_ and ambient O_2_ as a control. At days in vitro (DIV) 2, the medium was exchanged for complete feeding medium and experiments were performed on cultures at 9–11 DIV.

### Measurement of cellular oxygen consumption

Cellular oxygen consumption rate (OCR) and Extracellular Acidification rate (ECAR) were measured using a Seahorse XF^e^96 Extracellular Flux Analyzer (Seahorse Bioscience^26^) as previously described^16^. Primary cortical neurons were plated in XF^e^96 cell culture plates and maintained for 9–11 DIV at 37 °C, 5% CO_2_ and either in ambient (21%) or in 5% O_2_.

Prior to experimentation, the cells were equilibrated with XF Base Medium, bicarbonate-free DMEM-based medium (without pyruvate, lactate, glucose or glutamine) supplemented with L-lysine (0.136 mM) and L-arginine (0.574 mM) and pH 7.4, for 1 h. After baseline measurements, cells were either stimulated with glucose (15 mM or 2.5 mM, gluc) or with 3-beta hydroxybutyrate (5 mM, BHB) as solo substrates or in combination with glutamate (100 μM).

Substrates and glutamate, prepared in the same medium in which the experiment was conducted, were automatically injected from the first port, A, to the wells at the times indicated. Calibration of the respiration took place after the injection from port A. Mitochondrial function in neurons was determined through sequential addition of 3 μM oligomycin (Oli), 0.5 μM Carbonyl cyanide-4-(trifluoromethoxy)phenylhydrazone (FCCP), and 1 μM antimycin A / 1 μM rotenone (A/R). This allowed the determination of basal oxygen consumption, oxygen consumption linked to ATP synthesis (ATP), non-ATP linked oxygen consumption (proton leak), mitochondrial uncoupled respiration (MUR), and non-mitochondrial oxygen consumption (NM)^26–28^. Basal respiration was calculated by subtracting non-mitochondrial respiration from OCR after the initial stabilization (third measurement), and was considered 100%. Glycolytic capacity was measured as the ECAR rate reached by a given cell after the addition of the substrate with or without glutamate stimulation. Basal acidification state was considered as 100% and calculated after ECAR stabilization (third measurement).

BAPTA-AM (50 μM) was loaded into the cells using Ca^2+^-free Krebs buffer (140 mM NaCl, 5.9 mM KCl, 1.2 mM MgCl_2_, 15 mM HEPES, with 15 mM glucose and 1% B27) for 60 min. Afterwards, the cells were washed and equilibrated in experimental XF Base Medium (with no carbon sources and 1.8 mM CaCl_2_) for 15 min before starting the experiment.

Cortical neurons were treated with 0.5 mM aminooxyacetic acid (AOAA) for 1 h in feeding medium prior to experimentation. Afterwards, the cells were washed and equilibrated in experimental XF Base Medium (with no carbon sources and 1.8 mM CaCl_2_) for 15 min before starting the experiment. It is important to note that, in spite of the fact that neurons were grown at 5% O_2_ for 9–10 days, the bioenergetic experiments were performed in ambient conditions since the Seahorse XF^e^96 Extracellular Flux Analyzer could not be placed inside the BioSpherix Xvivo × 3 hypoxia chamber.

### Imaging of spontaneous cytosolic calcium oscillations in primary hippocampal neurons

At DIV 9–10, primary hippocampal neurons were loaded with Fluo-4 AM (6 μM) in fresh pre-warmed Krebs buffer (140 mM NaCl, 5.9 mM KCl, 1.2 mM MgCl_2_, 15 mM HEPES and 2.5 mM CaCl_2_, with 15 mM glucose or 5 mM BHB, pH 7.4), for 30 min. Neurons were then washed in fresh, pre-warmed buffer and placed on the stage of an LSM 5 Live confocal microscope in a thermostatically-regulated stage incubator set at 37 °C, 5% CO_2_ and an O_2_ concentration controller set to concentrations as indicated (Carl Zeiss and Pecon, both Germany). Single-cell imaging was performed using a 40 × , 1.3 NA oil-immersion objective (Carl Zeiss) run with ZEN software (Carl Zeiss, UK).

Fluo-4 was excited at 488 nm and the emission was collected through a 505–550 nm barrier filter. All microscope settings including laser intensity, scan time and image rate (300 frames/min) were kept constant for the whole set of experiments. After 2 min baseline recording of spontaneous neuronal activity (600 cycles), the buffer was exchanged for pre-warmed magnesium-free Krebs buffer (140 mM NaCl, 5.9 mM KCl, 15 mM HEPES and 2.5 mM CaCl_2_) with the corresponding substrate (15 mM glucose or 5 mM BHB) and equilibrated with the previously used O_2_ concentration. Then, the cells were imaged for a further 2 min (cycle 600–1200), before bolus addition of MgCl_2_ (1.2 mM final concentration) and recording for another two minutes. All images were processed and analysed using ImageJ 1.51 k Software (Wayne Rasband, NIH, USA). The single cell, intracellular Fluo-4 kinetics were analysed for spike frequency, total number of spikes and area under the curve. All single cell kinetics presented are normalized to their respective baseline values.

### Imaging of cytosolic calcium and mitochondrial membrane potential

Primary cortical neurons were loaded with Fluo-4 AM (6 μM) for 30 min in feeding medium. The neurons were then washed in fresh pre-warmed experimental Krebs buffer (as above) and subsequently incubated with TMRM (10 nM) in experimental Krebs buffer for 30 min prior to experimentation. The cortical neurons were placed on the stage of an LSM 710 confocal microscope equipped with a 40 × , 1.3 NA oil-immersion objective, a thermostatically regulated chamber set at 37 °C, 5% CO_2_, and an O_2_ concentration controller (Carl Zeiss and Pecon). TMRM was excited at 561 nm, and emission was detected in the range of 562—710 nm. Fluo-4 was excited at 488 nm, and the emission was detected in the range of 489–552 nm. All microscope settings including laser intensity and scan time were kept constant for the whole set of experiments, taking images every 60 s. After a baseline equilibration time, glutamate (100 μM final concentration) was added to the medium. All images were processed and analyzed using ImageJ 1.51 k Software, and the data presented were normalized to the baseline.

### Statistical analysis

Data are given as means ± SEM. Data were analysed using one-way ANOVA, followed by Tukey's post hoc test or Student's t test for two-group comparison. *p* values < 0.05 were considered to be statistically significant. All statistical analyses were performed using GraphPad Prism 5.

## Results

### BHB reduces spontaneous neuronal activity in neurons cultured in physiological oxygen levels

**Figure 1:**
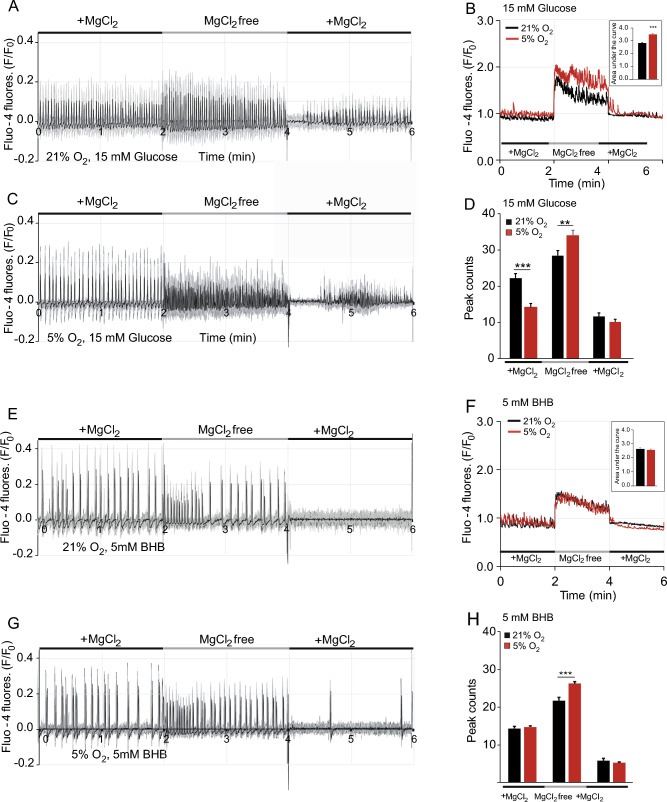
Spontaneous neuronal activity in primary neurons modified by physiological O_2_ and BHB. (**A**–**D**) In the presence of 15 mM glucose as a substrate**,** spontaneous cytosolic Ca^2+^ oscillations were observed in Fluo-4 loaded hippocampal neurons cultured and imaged at 21% O_2_ (**A**) and 5% O_2_ (**C**) concentrations, together with an increase in frequency and intensity after external Mg^2+^ withdrawal (MgCl_2_ free) and a subsequent recovery after Mg^2+^ addition. The area under the curve (**B**) and number of peaks (**D**) were analyzed**.** Data represent mean ± SEM from 3–4 independent experiments and a total of 157–190 regions of interest (ROIs). Traces from individual ROIs are in grey, with the average fluorescence intensity in each condition represented in bold black. Statistical analysis for two group comparison was assessed with two-tailed unpaired Student's t test. **p < 0.01, ***p < 0.001. In the presence of 5 mM 3-β OH butyrate (BHB) as a substrate (**E**–**H**), spontaneous cytosolic Ca^2+^ oscillations were observed in Fluo-4 loaded hippocampal neurons at 21% O_2_ (**E**) and at 5% O_2_ (**G**) concentration, together with an increase in frequency and intensity after external Mg^2+^ withdrawal. The area under the curve (**F**) and the number of peaks (**H**) were analyzed**.** Data represent mean ± SEM from 3–4 independent experiments and a total of 170–238 regions of interest (ROI) analysed.

We started our investigation by comparing neuronal Ca^2+^ signalling in primary neuron cultures maintained either at physiological O_2_ (5%) or ambient O_2_ (21%). Cytosolic Ca^2+^ oscillations are important characteristics of intact neuronal and network activity (see review written by Kar et al.^29^). We studied spontaneous Ca^2+^ oscillations at physiological and atmospheric O_2_ levels in Fluo-4 loaded primary hippocampal neurons in live time-course experiments and maintained in glucose. After 9–10 days of culture, axons and dendrites of the hippocampal neurons had developed into a sparse network that primarily formed an in vitro neural network. The hippocampal neurons were randomly selected for cytoplasmic Ca^2+^ analysis and the number of Ca^2+^ peaks and normalised fluorescence intensity were calculated. We observed typical Ca^2+^ oscillations in neurons cultured in both 21% and 5% O_2_ concentrations, and a similar normalized F/F_0_ Fluo-4 fluorescence intensity at baseline (Fig. 1A,C,E,G). The increase in fluorescence intensity after removal of Mg^2+^ from the media (MgCl_2_ free), which precludes Mg^2+^ ions from binding NMDA receptor pores thus facilitating intracellular Ca^2+^ entry, was significantly higher in neurons at physiological O_2_ concentration (Fig. 1B, Table 1) but not in BHB (Fig. 1F). Fluo-4 fluorescence recovered after Mg^2+^ re-addition both in ambient and in 5% O_2_ (Fig. 1A,C,E,G). The frequency in spontaneous synchronous cytosolic Ca^2+^ oscillations was determined by automatically counting cytosolic Ca^2+^ peaks (Fig. 1D,H)^30,31^. Interestingly, this revealed a significantly reduced basal frequency in neurons cultured in 5% O_2_ (22.13 ± 1.36 vs. 14.18 ± 0.99, *p* = 3.81 × 10^−6^) and a higher frequency during Mg^2+^ withdrawal (28.45 ± 1.41 vs. 33.95 ± 1.42, *p* = 0.0063) (Fig. 1D).

**Table 1:** Comparison of spontaneous activity in hippocampal neurons in the presence of glucose or BHB as substrates at 5% O_2_ or ambient conditions.

	Ambient	5% O_2_				
	MgCl_2_ free					
(A)						
Glucose (15 mM)	2.82 ± 0.07	3.50 ± 0.07				
BHB	2.65 ± 0.07	2.58 ± 0.06***				

	Ambient	5% O_2_	Ambient	5% O_2_	Ambient	5% O_2_
	+ MgCl_2_ (1)		MgCl_2_ free (2)		+ MgCl_2_ (3)	
(B)						
Glucose (15 mM)	22.13 ± 1.36	14.18 ± 0.99	28.45 ± 1.41	33.95 ± 1.42	11.50 ± 0.96	9.78 ± 0.94
BHB	14.15 ± 0.58***	14.54 ± 0.36	21.53 ± 0.80***	26.08 ± 0.47***	5.58 ± 0.66***	5.05 ± 0.22***

(A) Area under the curve calculations. (B) Peak counts in the three different sections of the experimental procedure, i.e., in basal state (1), after external magnesium withdrawal (2) and after magnesium re-addition (3). All data represent mean values ± SEM from 3–4 independent experiments, in a total of 157–238 regions of interest (ROIs). Statistical significance was assessed with two-tailed unpaired Student's t test. In A, ***p < 0.001, comparing 15 mM glucose at 5% O_2_ versus BHB at 5% O_2_In B, Peak counts: ***p < 0.001, comparing 15 mM glucose versus BHB at different O_2_ concentrations and specifies significant down-regulation.

**Figure 2:**
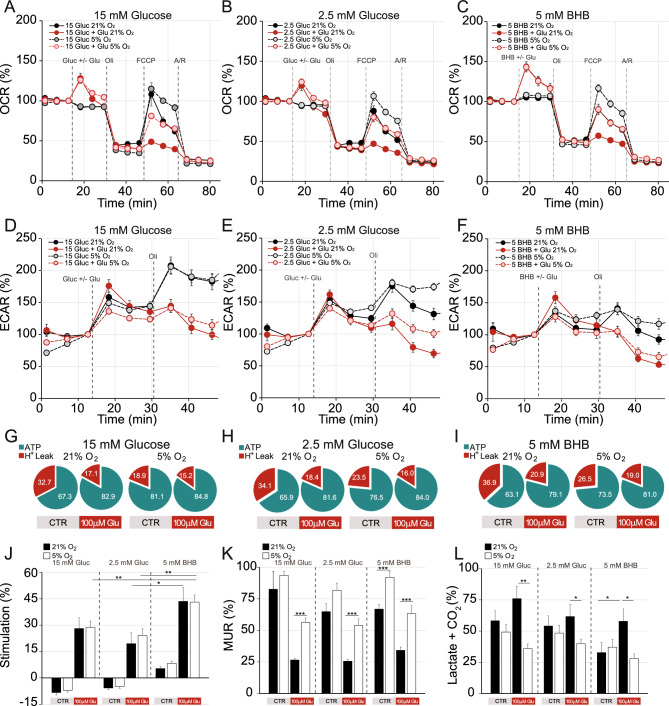
Maximal uncoupled respiration and the percentage of O_2_ consumption linked to ATP synthesis are increased, while the stimulation of glycolysis is reduced at physiological O_2_ (5%) compared to ambient O_2_ (21%). Cellular oxygen consumption rate (OCR) (**A**–**C**) and extracellular acidification rate (ECAR) (**D**–**F**) were measured using a Seahorse XF^e^96 Extracellular Flux Analyzer (Seahorse Bioscience^26^**.** Sequential injection of substrate (15 mM glucose **A** and **D**; 2.5 mM glucose **B** and **E**; 5 mM BHB **C** and **F**) with or without 100 μM glutamate (Glu), and metabolic inhibitors oligomycin (Oli, 6 μM), carbonyl cyanide-4-(trifluoromethoxy) phenylhydrazone (FCCP, 0.5 μM) and antimycin A/rotenone (Ant/Rot, 1 μM/1 μM) at different time points as indicated with dashed lines, enabled determination of bioenergetic parameters. ATP synthesis and proton (H^+^) leak parameters were calculated for neurons cultured in ambient (21%) and 5% O_2_ concentration using 15 mM glucose (**G**); 2.5 mM glucose (**H**); or 5 mM 3-beta OH butyrate (BHB) (**I**) in the control condition (CTR) and after glutamate stimulation (100 μM Glu). Basal respiration was calculated subtracting non-mitochondrial respiration from OCR after the initial stabilization (third measurement), and it was considered 100%. The percentage of stimulation of mitochondrial respiration (**J**), maximal uncoupled respiration (MUR) (**K**) and lactate secretion + CO_2_ production (**L**) in basal state (non-stimulated conditions) and in response to 100 μM glutamate were calculated in relation to basal OCR and determined in the presence of glucose or BHB as indicated in the figure. Glycolytic capacity was measured as the ECAR rate reached by a given cell after the addition of the substrate with or without glutamate stimulation. Basal acidification state was considered as 100% and calculated after ECAR stabilization (third measurement). All data represent mean values ± SEM from 3–4 independent experiments. Statistical analysis was assessed using a two-tailed unpaired Student's t test, * *p* < 0.05; **p < 0.01; *** *p* < 0.001.

Interestingly, in the presence of BHB, the OCR involved in ATP synthesis was significantly higher in cortical neurons grown in physiological O_2_ than in ambient conditions (63.11 ± 4.11% vs. 73.48 ± 2.80% in 5 mM BHB at ambient and 5% O_2_ respectively, *p* = 0.046, Fig. 2I). This was also similar to that observed in the presence of glucose as substrate (Fig. 2G,H). The mitochondrial respiratory control ratio was defined as state 3 respiration during highest ATP consumption divided by state 4 respiration with ATP synthase inhibited by Oligomycin^27^. This is calculated as the ratio of the respiration supporting ATP synthesis (OXPHOS, defined by the difference induced through inhibition of ATP synthase with Oligomycin, μATP) to the respiration ‘wasted’ to offset the proton leak (proton H^+^ leak) defined with baseline consumption subtracted by the remaining OCR after block of the respiratory chain with Antimycin and Rotenone. In cortical neurons the mitochondrial respiratory control ratio was lower (*p* = 0.0103, Table 2) in 5 mM BHB at ambient and 5% O_2_ respectively (Table 2), indicating highly coupled and efficient mitochondria in physiological conditions. Moreover, the MUR was significantly enhanced at physiological O_2_ concentration in unchallenged neurons in 5 mM BHB (Fig. 2K), which suggests an increased intrinsic respiratory capacity at 5% O_2_ using BHB as substrate.

**Table 2:** Summary of bioenergetic parameters.

		Stimulation (%)		ATP/H^+^Leak		MUR (%)		Glycolysis (%)	
		21% O_2_	5% O_2_	21% O_2_	5% O_2_	21% O_2_	5% O_2_	21% O_2_	5% O_2_
Glucose (15 mM)	Control	− 8.31 ± 1.38	− 7.07 ± 1.67	2.49 ± 0.27	4.38 ± 0.17	82.63 ± 14.15	93.58 ± 3.26	50.24 ± 8.10	49.18 ± 6.37
	+ Glu	28.06 ± 6.15	26.04 ± 3.57	4.99 ± 0.29	5.66 ± 0.21	26.52 ± 1.21	56.27 ± 3.50	75.98 ± 9.87	39.05 ± 2.16
	+ Glu + AOAA	− 7.66 ± 2.37	− 1.35 ± 2.99	2.59 ± 0.11	3.44 ± 0.25	22.16 ± 1.21	46.29 ± 2.28	54.82 ± 5.64	57.23 ± 4.41
Glucose (2.5 mM)	Control	− 5.69 ± 1.98	− 4.87 ± 1.78	2.45 ± 0.29	3.88 ± 0.33	64.81 ± 6.38	81.74 ± 5.72	54.04 ± 8.86	48.36 ± 4.84
	+ Glu	19.48 ± 6.21	24.11 ± 3.81	4.60 ± 0.34	5.68 ± 0.49	25.48 ± 1.62	53.99 ± 5.22	61.67 ± 7.04	39.89 ± 3.95
	+ Glu + AOAA	− 4.91 ± 3.67	− 11.38 ± 4.11	2.13 ± 0.07*	2.49 ± 0.23*	30.91 ± 1.71^#^	33.84 ± 3.76*	38.22 ± 6.55	75.69 ± 10.72
BHB (5 mM)	Control	5.32 ± 1.08^#^	8.29 ± 1.28^#^	2.24 ± 0.30	3.41 ± 0.31*	66.84 ± 3.77	92.06 ± 4.83	32.76 ± 4.67*	37.07 ± 6.52
	+ Glu	43.54 ± 7.93^#^	43.08 ± 4.06^#^	3.82 ± 0.18*	4.73 ± 0.35*	34.17 ± 2.56^#^	63.28 ± 6.31	57.88 ± 9.49	28.01 ± 7.87
	+ Glu + AOAA	62.06 ± 7.58^#^	52.09 ± 5.53^#^	3.22 ± 0.40	3.53 ± 0.22	36.03 ± 3.38^#^	50.37 ± 5.81	34.64 ± 7.98	62.01 ± 14.58

Compilation of bioenergetic parameters in primary neuron cultures grown at 21% or 5% O_2_ concentration in control conditions, upon 100 μM glutamate stimulation (+ Glu) and after 1 h of aminooxyacetic acid treatment (AOAA, 0.5 mM) and subsequent 100 μM glutamate stimulation (+ Glu + AOAA). Statistic considerations are mentioned in the text. Percentages (%) are calculated according to OCR or ECAR prior to substrate addition.

*Specifies significant down-regulation.

^#^Significant up-regulation comparing 15 mM glucose versus 2.5 mM glucose or 5 mM BHB within the same treatment group and O_2_ concentration condition.

### Effect of BHB on glutamate-induced Ca^2+^ overloading

Having shown that in neurons using 2.5 mM or 15 mM glucose or 5 mM BHB, basal respiration was not limited by substrate supply, we next studied the control of respiration by agents able to increase neuronal workload at physiological O_2_ concentration. Oxygen consumption is controlled by the mitochondrial proton electrochemical gradient (∆μH^+^^32^). In most cell types, ∆μH^+^ is mainly used in ATP synthesis. Increases in cell workload will consume ATP and lead to increased ATP production in mitochondria, through the utilization of ∆μH^+^, which is expected to increase OCR. In particular, we studied the effects of glutamate-induced workload increase.

**Figure 3:**
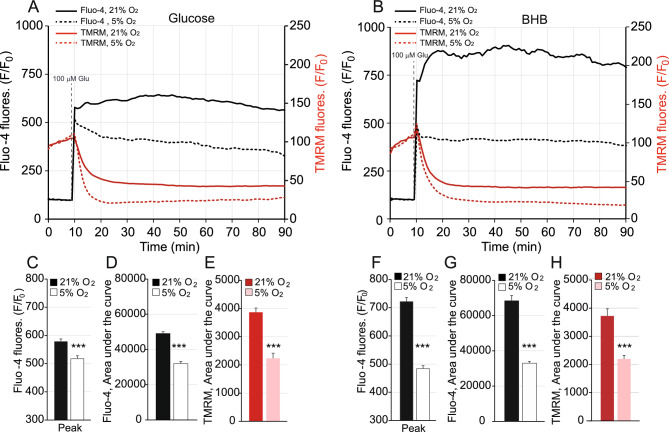
Lower cytosolic Ca^2+^ levels and earlier mitochondrial membrane potential decrease are observed at physiological O_2_ (5%) upon glutamate stimulation. Changes in cytosolic Ca^2+^ (black lines) and mitochondrial membrane potential (red lines) in Fluo-4 and TMRM loaded neurons respectively obtained by stimulation with 100 μM glutamate in 15 mM glucose (**A**, **C**–**E**) or in 5 mM 3-beta OH butyrate (BHB) (**B**, **F**–**H**) medium, in the presence of 2.5 mM Ca^2+^, at 21% O_2_ (solid lines) or 5% O_2_ (dashed lines) conditions. Normalised mean Fluo-4 and TMRM fluorescence from 2–3 independent experiments and a total of 144–338 cells are represented (**A**, **B**). Quantification of the cytosolic calcium peak upon glutamate stimulation was determined in ambient (black bar) and in 5% O_2_ (empty bar) in the presence of glucose (**C**) or 3-beta OH butyrate (**F**) as substrate. **D**–**H** represent the areas under the curve for simultaneous measurements of cytosolic calcium (**D**, **G**) and mitochondrial membrane potential (**E**, **H**) using glucose (**D**, **E**) or BHB (**G**, **H**) as substrate. All showed a significantly lower value at 5% O_2_.

Glutamate (Glu) is the main excitatory neurotransmitter of the central nervous system. In the presence of 15 mM glucose, glutamate addition induced a pronounced and sustained increase in cytosolic Ca^2+^ in neurons cultured in 21% O_2_, which was reduced in neurons cultured in physiological O_2_ (black dashed lines) (Fig. 3A). NMDA receptor activation induced early mitochondrial depolarization, shown by a decrease in tetramethylrhodamine methyl ester (TMRM) fluorescence (Fig. 3A), as previously described^33^. Interestingly, the lower cytosolic Ca^2+^ levels observed at 5% O_2_ were associated with a more pronounced decrease in mitochondrial membrane potential, which might reflect increased mitochondrial Ca^2+^ buffering capacity (Fig. 3A,C,D,E).

Similar effects were observed in the presence of BHB (Fig. 3B,F,G,H), although the glutamate-mediated peak in cytosolic Ca^2+^ in ambient conditions was significantly higher than that observed in neurons using glucose (578.64 ± 9.08 vs. 721.86 ± 14.39 normalized F/F_0_ fluorescence, with 15 mM glucose or 5 mM BHB in 21% O_2_ respectively, *p* = 1.135 × 10^−5^, Fig. 3C,F). Interestingly however, an even stronger attenuation of the cytosolic Fluo-4 fluorescence was registered at physiological O_2_ concentration in the presence of BHB (Fig. 3B,F) compared to glucose (Fig. 3A,C) (517.19 ± 10.47 vs. 438.95 ± 10.42 normalized F/F_0_ fluorescence, with 15 mM glucose or 5 mM BHB at 5% O_2_ respectively, *p* = 0.025).

### BHB increases glutamate-mediated stimulation of respiration and mitochondrial ATP production, and preserves maximal respiratory capacity

As dictated by the principles of chemiosmotic coupling, changes in workload after glutamate stimulation promoted increased mitochondrial ATP production (Fig. 2G–I) coupled to increased O_2_ consumption by the respiratory chain and increased substrate supply to mitochondria (Fig. 2A–C). The magnitude of the glutamate-mediated stimulation of OCR (Fig. 2J) was similar in neuronal cultures grown in ambient and at 5% O_2_, using 15 mM or 2.5 mM glucose. Interestingly, BHB was a more effective substrate, allowing significantly higher stimulation of mitochondrial respiration regardless of the O_2_ concentration in which primary neurons had grown (Fig. 2J).

Moreover, the measure of mitochondrial efficiency, the respiratory control ratio, was higher after glutamate stimulation than in control unchallenged conditions for all substrates and O_2_ conditions (Fig. 2G–I, Table 2). Importantly, the respiratory control ratio after glutamate injection was significantly larger in neurons cultured in 5% O_2,_ showing a higher capability to increase ATP production compared to neurons cultured in ambient conditions, only in the presence of BHB ( *p* = 0.0315, Fig. 2I, Table 2).

Interestingly, the inability of mitochondria to sustain glutamate-stimulated respiration in neurons cultured in ambient O_2_, reflected by the large decrease in maximal uncoupled respiration (MUR) regardless of the substrate present, strikingly contrasted with the effect observed at physiological O_2_ levels (Fig. 2K). At 5% O_2_ MUR was significantly preserved (Fig. 2K, Table 2). Thus, neurons grown at physiological O_2_ concentration might be protected from mitochondrial dysfunction induced by glutamate stimulation.

The glutamate-induced increase in workload promoted an increase in mitochondrial respiration and a limited rise in the glycolytic flux (Fig. 2D–F). Moreover, the glutamate-mediated stimulation of glycolysis was jeopardized at physiological O_2_ concentration, reaching lower values compared to ambient condition in the presence of either glucose or BHB as substrates (Fig. 2L).

### BHB’s effect on mitochondrial responses to glutamate stimulation is Aralar-MAS independent

**Figure 4:**
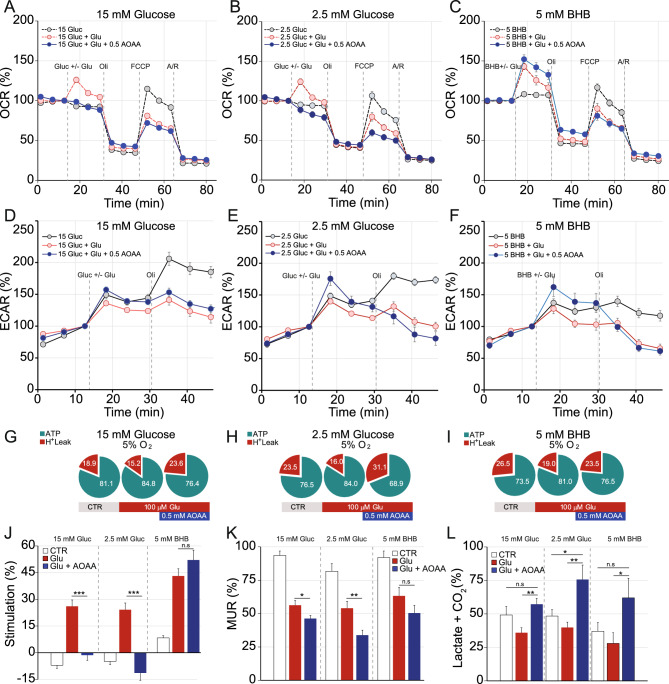
Malate-Aspartate Shuttle (MAS) inhibition does not affect bioenergetic profiles of cortical neurons cultured at 5% O_2_ using BHB, but does affect those using glucose. Cellular oxygen consumption rate (OCR) (**A**–**C**) and extracellular acidification rate (ECAR) (**D**–**F**) were measured in cortical primary neurons cultured at 5% O_2_ using a Seahorse XF^e^96 Extracellular Flux Analyzer (Seahorse Bioscience^26^. Sequential injection of substrate (15 mM glucose **A** and **D**; 2.5 mM glucose **B** and **E**; 5 mM BHB **C** and **F**) in control conditions or in the presence of 100 μM glutamate +/− 1 h incubation with the metabolic inhibitor aminooxyacetic acid (AOAA, 0.5 mM): oligomycin (Oli, 6 μM), carbonyl cyanide-4-(trifluoromethoxy) phenylhydrazone (FCCP, 0.5 μM) and antimycin A/rotenone (Ant/Rot, 1 μM/1 μM) at time points indicated with dashed lines. Respiratory parameters: ATP synthesis and proton leak using 15 mM glucose (**G**); 2.5 mM glucose (**H**); and 5 mM BHB (**I**); as well as stimulation of mitochondrial respiration upon substrate addition +/− glutamate (**J**), maximal uncoupled respiration (MUR) (**K**) and glycolysis (**L**) were determined in neurons cultured in 5% O_2_ after 1 h of AOAA treatment (aminooxyacetic acid, 0.5 mM). Glutamate-mediated stimulation of mitochondrial respiration was completely abolished after MAS inhibition in the presence of glucose (**A**, **B**, **J**) but preserved with BHB (**C**, **J**), together with an increase in lactate secretion + CO_2_ production (**L**). All data represent mean values ± SEM from 3–4 independent experiments. Statistical analysis was assessed using a two-tailed unpaired Student’s t test, *p < 0.05; **p < 0.01; ***p < 0.001.

We next studied the influence of Aralar/AGC1, the regulatory component of the malate-aspartate NADH shuttle (MAS), on glutamate excitotoxicity. We determined the effects of aminooxyacetic acid (0.5 mM AOAA), the inhibitor of aspartate aminotransferase (AST) which is a widely used inhibitor of the MAS^34^, on primary cortical neurons grown in 5% O_2_ (Fig. 4A–L) and in ambient conditions (Supplementary Fig. 1), with glucose or BHB as substrates. We found that the glutamate-induced increase in OCR observed with glucose as substrate was completely abolished by AOAA (Fig. 4A,B,J) indicating that Aralar-MAS has an essential role in this response. Interestingly, AOAA incubation did not abolish the glutamate-induced increase in respiration in neurons with BHB as substrate (Fig. 4C,J).

The Aralar-MAS transfers the reducing equivalents of cytosolic NADH into mitochondria, thus increasing mitochondrial ATP production^35,36^. Consistently, an increase in the glycolytic flux was observed in neurons cultured in 5% O_2_ and treated with AOAA in the presence of glucose, potentially indicating a compensatory mechanism to overcome the inefficient respiration upon glutamate stimulation to match the increased ATP demand (Fig. 4D,E,L). Interestingly, this significant increase in glycolysis was also observed in neurons treated with AOAA in the presence of BHB (Fig. 4F,L), although these neurons were able to promote an Aralar-independent stimulation of OXPHOS (Fig. 4F,L). Moreover, the described effects on glycolysis were only observed in neurons cultured at 5% O_2_, but not in cortical neurons grown in ambient conditions, in which the glutamate-mediated stimulation of glycolysis was not observed (see Supplementary Fig. 1D–F,L).

AOAA treatment compromises NADH supply to the respiratory chain, and this led to a reduction in mitochondrial efficiency particularly observed with glucose, determined by the decrease of the O_2_ consumption linked to ATP synthesis and an increase in proton leak compared to control (Fig. 4G–H). Thus, the mitochondrial respiratory control ratio (ATP synthesis/proton leak) in neurons with 15 mM glucose was: control 4.38 ± 0.17 versus glutamate + AOAA 3.34 ± 0.25 (*p* = 0.005), and in neurons with 2.5 mM glucose was: control 3.88 ± 0.33 versus glutamate + AOAA 2.49 ± 0.23 (*p* = 0.002, Table 2). Maximal uncoupled respiration (MUR) was also substantially affected by AOAA in neurons using glucose as a substrate, reaching even lower values compared to glutamate stimulation in the absence of AOAA (Fig. 4K). Neurons using 15 mM glucose exhibited MUR values of: with glutamate 56.27 ± 3.50% versus glutamate + AOAA 46.29 ± 2.28% (*p* = 0.025); and in the presence of 2.5 mM glucose MUR values were: with glutamate 53.99 ± 5.22% versus glutamate + AOAA 33.84 ± 3.76% (*p* = 0.004, Table 2).

No significant effects in the respiratory control ratio (control 3.41 ± 0.31 vs. glutamate + AOAA 3.53 ± 0.22, *p* = 0.760) or in MUR (with glutamate 63.28 ± 6.31% vs. glutamate + AOAA 50.37 ± 5.81%, *p* = 0.140) were found in the presence of BHB (Fig. 4I,K, Table 2). Taken together, these data suggest that, contrary to what was observed with glucose, BHB modulates mitochondrial respiration independently of Aralar-MAS activity in physiological O_2_ conditions.

### BHB effect on mitochondrial function upon glutamate stimulation relies on cytosolic calcium signalling

Glutamate-mediated NMDA receptor activation causes Na^+^ and Ca^2+^ entry into the neuronal cytosol, which induces the activation of plasma membrane and endoplasmic reticulum ATPase pumps to restore the intracellular ionic balance while consuming a vast amount of ATP^37^. BAPTA-AM is a rapid intracellular Ca^2+^-chelator^38^. To investigate whether cytosolic Ca^2+^ signalling may mediate BHB effects on mitochondrial function during glutamate stimulation, primary cortical neurons were incubated with BAPTA-AM and then challenged with glutamate.

**Figure 5:**
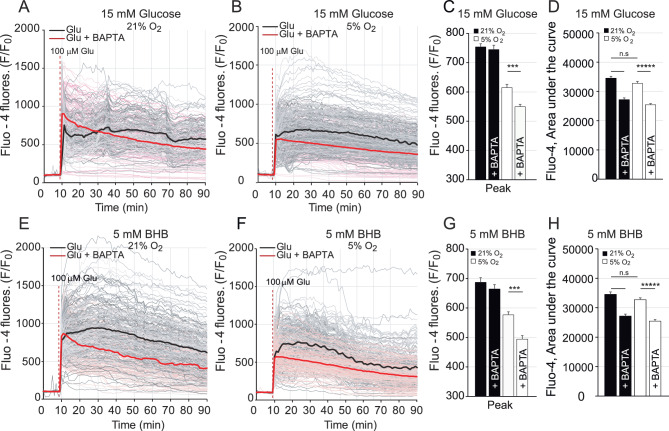
Glutamate-mediated cytosolic Ca^2+^ response is reduced in primary cortical neurons in the presence of the Ca^2+^ chelator BAPTA. Changes in cytosolic Ca^2+^ in Fluo-4 loaded neurons obtained by stimulation with 100 μM glutamate after 1 h of BAPTA treatment (cytosolic calcium chelator tetraacetoxymethyl ester, 50 μM) in ambient (**A**, **E**) or in 5% O_2_ (**B**, **F**) conditions using 15 mM glucose (**A**–**D**) or 5 mM BHB (**E**–**H**) as solo substrate. Recordings from individual cells (gray and light red) and average (black and red) are shown from 4 independent experiments with a total of 183–380 cells. Quantification of the cytosolic calcium peak (**C**, **G**) and the area under the curve (**D**, **H**) upon glutamate stimulation was determined in ambient (black bars) and in 5% O_2_ (empty bars) after 1 h of BAPTA treatment when indicated.

**Figure 6:**
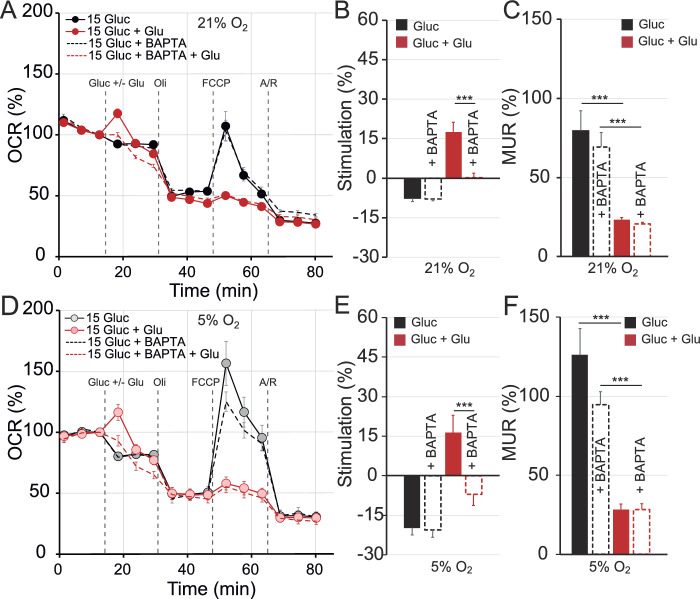
Ca^2+^ chelator BAPTA treatment jeopardizes respiratory response in neurons cultured in ambient and at 5% O_2_ concentration using glucose. Cellular oxygen consumption rates (OCR) (**A**, **D**) were measured in cortical primary neurons cultured in ambient (**A**) and at 5% O_2_ (**D**) using a Seahorse XF^e^96 Extracellular Flux Analyzer (Seahorse Bioscience^26^. Analyser was set in ambient conditions. Sequential injection of substrate (15 mM glucose) in control conditions or in the presence of 100 μM glutamate (Glu) and metabolic inhibitors: oligomycin (Oli, 6 μM), carbonyl cyanide-4-(trifluoromethoxy) phenylhydrazone (FCCP, 0.5 μM) and antimycin A/rotenone (Ant/Rot, 1 μM/1 μM) at time points indicated with dashed lines. Respiratory parameters: stimulation of mitochondrial respiration (**B**, **E**) and, maximal uncoupled respiration (MUR) (**C**, **F**) were determined in neurons cultured at 21% O_2_ (**A**–**C**) or at 5% O_2_ (**D**–**F**) after 1 h of tetraacetoxymethyl ester treatment (BAPTA, 50 μM) when indicated. All data represent mean values ± SEM from 3–4 independent experiments. Statistical analysis was assessed with two-tailed unpaired Student's t test, * *p* < 0.05; **p < 0.01; *** *p* < 0.001.

In BAPTA-AM loaded neurons, glutamate-mediated Ca^2+^ signals in the cytosol were significantly reduced both at 5% and 21% O_2_ concentrations using glucose (Fig. 5A–D) or BHB (Fig. 5E–H) as substrates, without affecting basal respiration (not shown). With glucose as the substrate, glutamate–induced stimulation of OCR was severely decreased by BAPTA incubation in neurons cultured at either 5% or 21% O_2_ (Fig. 6A,B,D,E), while the glutamate-mediated decrease in MUR was unaffected by BAPTA-AM incubation (Fig. 6C,F). These results indicated that Ca^2+^-signalling is required for the glutamate-induced stimulation of respiration in neurons utilising glucose as substrate, and that the O_2_ concentration in which neurons are grown does not modulate this effect.

**Figure 7:**
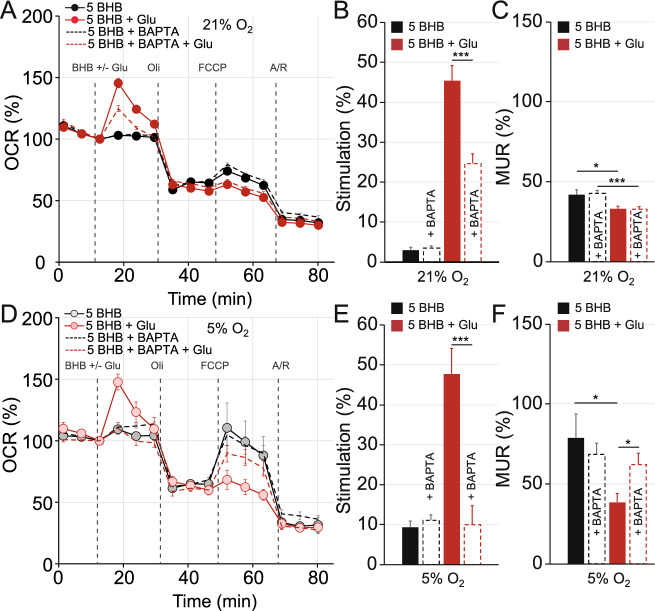
Ca^2+^ chelator BAPTA treatment partially prevents glutamate-mediated stimulation of respiration in ambient conditions, and preserves mitochondrial maximal respiratory capacity at 5% O_2_ concentration in cultured neurons using BHB. Cellular oxygen consumption rates (OCR) (**A**, **D**) were measured in cortical primary neurons cultured in ambient (**A**) and at 5% (**D**) O_2_ levels, using a Seahorse XF^e^96 Extracellular Flux Analyzer (Seahorse Bioscience^26^). Sequential injection of substrate (BHB, 5 mM) in control conditions or in the presence of 100 μM glutamate (Glu) and metabolic inhibitors: oligomycin (Oli, 6 μM), carbonyl cyanide-4-(trifluoromethoxy) phenylhydrazone (FCCP, 0.5 μM) and antimycin A/rotenone (Ant/Rot, 1 μM/1 μM) at time points indicated with dashed lines. Respiratory parameters: stimulation of mitochondrial respiration (**B**, **E**) and, maximal uncoupled respiration (MUR) (**C**, **F**) were determined in neurons cultured in 21% O_2_ (**A**–**C**) or in 5% O_2_ (**D**–**F**) after 1 h of tetraacetoxymethyl ester treatment (BAPTA, 50 μM) when indicated. All data represent mean values ± SEM from 3 to 4 independent experiments. Statistical analysis was assessed using a two-tailed unpaired Student’s t test, * *p* < 0.05; **p < 0.01; *** *p* < 0.001.

Interestingly, BAPTA-AM reduced, but did not eliminate, glutamate-mediated stimulation of OCR (glutamate 45.44 ± 3.75% vs. glutamate + BAPTA 24.69 ± 2.43%, *p* = 3.00 × 10^−5^) in neurons grown in ambient O_2_ and with BHB as substrate (Fig. 7A,B). This BAPTA-mediated effect was stronger in neurons grown at physiological O_2_ concentration, however, with a decrease in glutamate-induced stimulation up to 78.98% (with 5 mM BHB: glutamate 47.67 ± 6.49% vs. glutamate + BAPTA 10.02 ± 4.72%, *p* = 1.20 × 10^−4^ Fig. 7D,E) reaching levels comparable to OCR in control conditions (9.36 ± 1.53% at 5% O_2_). Moreover, MUR in BAPTA-AM preloaded neurons was preserved even after glutamate stimulation at 5% O_2_ (Fig. 7F), but not in ambient conditions (Fig. 7C), reaching levels similar to control with BHB.

These results suggest that primary cortical neurons rely on cytosolic Ca^2+^ signalling to induce glutamate-mediated OCR stimulation in the presence of glucose. However, with BHB as substrate, this cytosolic Ca^2+^ signalling pathway is only partially involved in the regulation of mitochondrial respiration in ambient conditions, and it is completely absent at 5% O_2_.

### BHB-mediated intracellular stored calcium mobilization upon glutamate is significantly reduced in cultures maintained in physiological oxygen concentration

**Figure 8:**
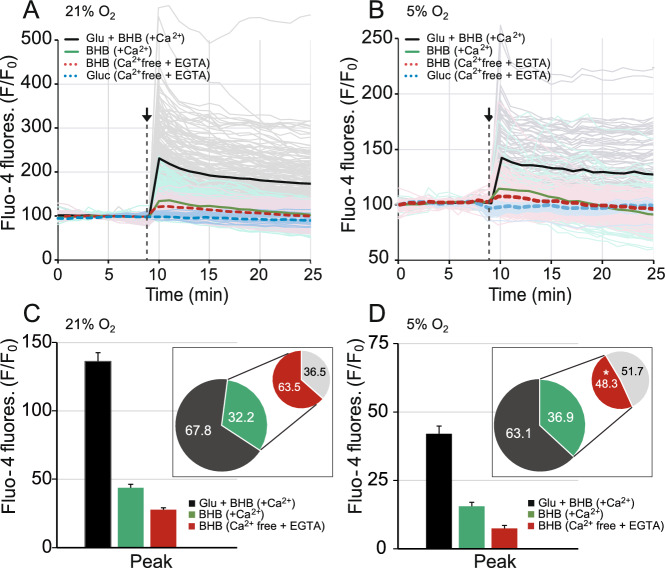
Intracellular stored Ca^2+^ mobilization upon glutamate stimulation is reduced in neurons cultured at physiological O_2_ concentration using BHB. Changes in cytosolic calcium in Fluo-4 loaded neocortical primary neurons obtained by stimulation with: (i) 5 mM BHB and 100 μM glutamate in the presence of 2.5 mM CaCl_2_ (Glu + BHB, black traces), (ii) 5 mM BHB without glutamate in the presence of 2.5 mM CaCl_2_ (BHB, green traces), (iii) 5 mM BHB without glutamate in the absence of CaCl_2_ plus 100 μM EGTA (BHB, red dashed traces) and (iv) 15 mM glucose without glutamate in the absence of CaCl_2_ plus 100 μM EGTA (Gluc, blue dashed traces). Normalised mean Fluo-4 fluorescence from 4 independent experiments and a total of 143–176 cells are represented in ambient (**A**) and from 3 independent experiments and a total of 104–122 cells are represented at physiological oxygen concentration (**B**); considering glucose-mediated stimulation as a negative control condition, studied in 1–2 independent experiments in a total of 43–55 cells. Quantification of the cytosolic Ca^2+^ peak upon the aforementioned conditions was performed in ambient (**C**) and in 5% O_2_ conditions (**D**). Graphical inserts in C and in D represent the calculations of the percentage of the signal considering condition (i) as the reference value in media with Ca^2+^, and condition (ii) as the reference to study BHB effects with and without Ca^2+^. All data represent mean values ± SEM. Statistical analysis was assessed with two-tailed unpaired Student’s t test, *p < 0.05.

Finally, we studied the source of Ca^2+^ which contributed to the glutamate-mediated increase in cytosolic Ca^2+^. Cytosolic Ca^2+^ levels were measured in neurons loaded with the fluorescent probe Fluo-4 in four different conditions in ambient (Fig. 8A) or at physiological O_2_ concentration (Fig. 8B): (i) 5 mM BHB with 100 μM glutamate in the presence of 2.5 mM CaCl_2_ (Glu + BHB, black traces), (ii) 5 mM BHB without glutamate in the presence of 2.5 mM CaCl_2_ (BHB, green traces), (iii) 5 mM BHB without glutamate in the absence of CaCl_2_ plus 100 μM EGTA (BHB, red dashed traces) and (iv) 15 mM glucose without glutamate in the absence of CaCl_2_ plus 100 μM EGTA (Gluc, blue dashed traces) used as negative control.

Lower cytosolic Fluo-4 fluorescence peaks (normalised F/F_0_) were registered at 5% O_2_ (Fig. 8C) compared to ambient O_2_ (Fig. 8D), as previously observed in an independent set of experiments (Fig. 5). Interestingly, we observed that BHB by itself stimulated an increase in cytosolic Ca^2+^ (green trace, Fig. 8A,B). Considering the first condition, Glu + BHB, as the reference value we calculated the percentage of cytosolic Ca^2+^ mobilized only by BHB in the presence of extracellular Ca^2+^. Thus, no statistical differences were found in BHB-mediated increase in cytosolic Ca^2+^ (in 21% O_2_ 32.15 ± 1.87% vs. 5% O_2_ 36.90 ± 3.53%, *p* = 0.237, graphical insert in C and in D in green).

Moreover, to analyse the source of Ca^2+^ in the BHB-mediated mobilization process we utilized media with 2.5 mM CaCl_2_ or Ca^2+^-free media in the presence of EGTA (100 μM), and the second condition (BHB in green) was used as the reference value to calculate the next percentages. We identified a significantly reduced contribution of Ca^2+^ from intracellular stores to promote the cytosolic Ca^2+^ rise in physiological O_2_ concentration (in 21% O_2_ 63.52 ± 3.02% vs. 5% O_2_ 48.26 ± 6.80%, *p* = 0.042). These results indicate that in our experimental conditions BHB-mediated intracellular stored Ca^2+^ mobilization upon glutamate was significantly reduced at physiological O_2_ concentration, a Ca^2+^ signalling mechanism apparently essential to promote mitochondrial respiration.

## Discussion

We here addressed how β-hydroxybutyrate (BHB), the main ketone body and energy substrate endogenously produced in a KD, affects mitochondrial bioenergetics during glutamate-mediated Ca^2+^ signalling in primary cultured neurons grown at in vivo O_2_ levels, and 2) which Ca^2+^-mediated mechanisms are involved in this process. BHB increased glutamate-mediated stimulation of respiration compared to glucose, and enabled stronger and more sustained maximal uncoupled respiration. Finally, these effects were independent of the malate-aspartate NADH shuttle activity, but relied on cytosolic Ca^2+^-dependent mechanisms.

### BHB improves mitochondrial energetics

In the present study, we aimed to assess the therapeutic effectiveness of BHB, the most abundant ketone body in mammals, in non-stimulated cortical neurons at physiological O_2_ concentration, and under glutamate-mediated excitotoxicity conditions relevant to epileptic seizures. BHB was mainly consumed by OXPHOS, since the injection of the ketone body was accompanied by a rise in respiration (Fig. 2C,J). At 5% O_2_ BHB increased MUR (Fig. 2C,K), an effect not observed with glucose, indicating the possibility that the ketone body is a more efficient substrate since MUR is mainly controlled by substrate supply, together with the intrinsic respiratory capacity of mitochondria^27^.

We also studied the contribution of Aralar-MAS activity on the glutamate-mediated increase in OCR in the presence of aminooxyacetic acid (0.5 mM AOAA), the inhibitor of AST, which is a widely used inhibitor of MAS^34^. Glutamate stimulation promoted an increase in cytosolic Ca^2+^ concentration which results in a net increase in ATP consumption in order to restore the cytosolic ionic balance^15^. Importantly, we identified BHB as a much more effective substrate than glucose in conditions of glutamate stimulation, allowing significantly higher and sustained stimulation of mitochondrial respiration (Fig. 4C,J) with a significant increase in O_2_ consumption linked to ATP synthesis observed at 5% O_2_ compared to 21% O_2_ (Fig. 4I). This indicates that varying substrates may enable mitochondria to face hyperexcitation more efficiently. Moreover, culturing in 5% O_2_ enabled the neurons to maintain their MUR in the presence of glutamate regardless of the substrate present, unlike those cultured in ambient conditions (Fig. 4K).

Previous work revealed that the lack of ARALAR-MAS prevented adequate glucose-derived pyruvate supply to the mitochondria, inducing a metabolic limitation^16^. Moreover, BHB was found to promote efficient recovery from deficits in both basal and glutamate-stimulated respiration in *aralar*-deficient neurons^39^. Here, our analysis performed in physiological O_2_ concentration reinforces the concept that ARALAR-MAS plays an essential role in the response to glutamate-induced increases in neuronal workload, and is a key mechanism for upregulating respiration in the presence of glucose, facilitating adequate pyruvate supply to the mitochondria (Fig. 4A,B,J). Moreover, BHB constitutes an effective substrate enabling primary cortical neurons to bypass energetic limitations imposed by ARALAR-MAS inhibition (Fig. 4C,J).

Interestingly, BHB-mediated Ca^2+^ mobilization (Fig. 8) and the effects on mitochondrial respiration upon glutamate stimulation seem to be significantly altered in cortical neurons in physiological O_2_ concentration (Fig. 7). In our experimental conditions, primary cortical neurons rely on cytosolic Ca^2+^ signalling to induce glutamate-mediated OCR stimulation in the presence of BHB (Fig. 7D,E). In ambient conditions, however, the presence of BAPTA-AM, a rapid intracellular Ca^2+^-chelator, only halved OCR stimulation (Fig. 7A,B). This effect might be mediated by BHB-induced ER Ca^2+^ release in the mitochondria-associated membranes (MAMs) points of contact between the ER and mitochondria, the signal which stimulates OXPHOS. Furthermore, BHB-mediated mobilisation of intracellular stored Ca^2+^ upon glutamate was significantly reduced at physiological O_2_ concentration (Fig. 8). One limitation of this work was that we evaluated short-term BHB-mediated signalling mechanisms induced by acute administration of the ketone body, relevant to the seizure setting. However, these short-term effects may need to be dissected from long-term effects that involve modulation in gene expression in physiological O_2_ concentrations.

## Conclusion

Together these results underscore the role of BHB as a more efficient substrate to sustain mitochondrial respiration upon glutamate-mediated stimulation at physiological O_2_ concentrations compared to glucose. Moreover, BHB-mediated cytosolic calcium signalling is required to fully stimulate mitochondrial respiration. Collectively, the experimental evidence provided suggests that ketone administration alone might afford anti-seizure benefits for patients with epilepsy by enhancing mitochondrial function.

### Supplementary Information

Supplementary Information.

Supplementary Figure S1.

## Data Availability

The datasets used and/or analysed during the current study available from the corresponding author on reasonable request.
