# Supplementary material for: Ketone body β-hydroxybutyrate (BHB) preserves mitochondrial bioenergetics
Source: Sci Rep. 2023 Nov 11;13:19664. doi: 10.1038/s41598-023-46776-8 (PMC10640643; doi:10.1038/s41598-023-46776-8)
Supplement: Supplementary file 1 — Supplementary Information. [file 41598_2023_46776_MOESM1_ESM.docx]

**SUPPLEMENTARY FIGURE LEGENDS**

**Supplementary Figure 1. Malate-Aspartate Shuttle (MAS) inhibition does not affect bioenergetic profiles of cortical neurons cultured at 21% O_2_** **using BHB but does in those using glucose.**

Cellular oxygen consumption rate (OCR) (**A-C**) and extracellular acidification rate (ECAR) (**D-F**) were measured using a Seahorse XF^e^96 Extracellular Flux Analyzer (Seahorse Bioscience). Sequential injection of substrate (15 mM glucose **A** and **D**; 2.5 mM glucose **B** and **E**; 5 mM BHB **C** and **F**) in control conditions or in the presence of 100 μM glutamate and metabolic inhibitors to perform the calibration of the respiration: oligomycin (Oli, 6 μM), carbonyl cyanide-4-(trifluoromethoxy) phenylhydrazone (FCCP, 0.5 μM) and antimycin A/rotenone (Ant/Rot, 1 μM/1 μM) at time points indicated with dashed lines. Respiratory parameters: ATP synthesis and proton leak using 15 mM glucose (**G**); 2.5 mM glucose (**H**); and 5 mM BHB (**I**); stimulation of mitochondrial respiration **(J)**, maximal uncoupled respiration (MUR) **(K)** and lactate secretion + CO_2_ production **(L)** were determined in neurons growing at 21% O_2_ after 1 hour of AOAA treatment (aminooxyacetic acid, 0.5 mM). Glutamate-mediated stimulation of mitochondrial respiration was completely abolished after MAS inhibition in the presence of glucose (**A, B, J**) but preserved with BHB (**C, J**), together with an increase in glycolysis (**L**). All data represent mean values ± SEM from 3-4 independent experiments. Statistical analysis was assessed with two-tailed unpaired Student's t test, * p<0.05; **p<0.01; *** p<0.001.
