# Supplementary figures and images for: Ketone body β-hydroxybutyrate (BHB) preserves mitochondrial bioenergetics
Source: Sci Rep. 2023 Nov 11;13:19664. doi: 10.1038/s41598-023-46776-8 (PMC10640643; doi:10.1038/s41598-023-46776-8)

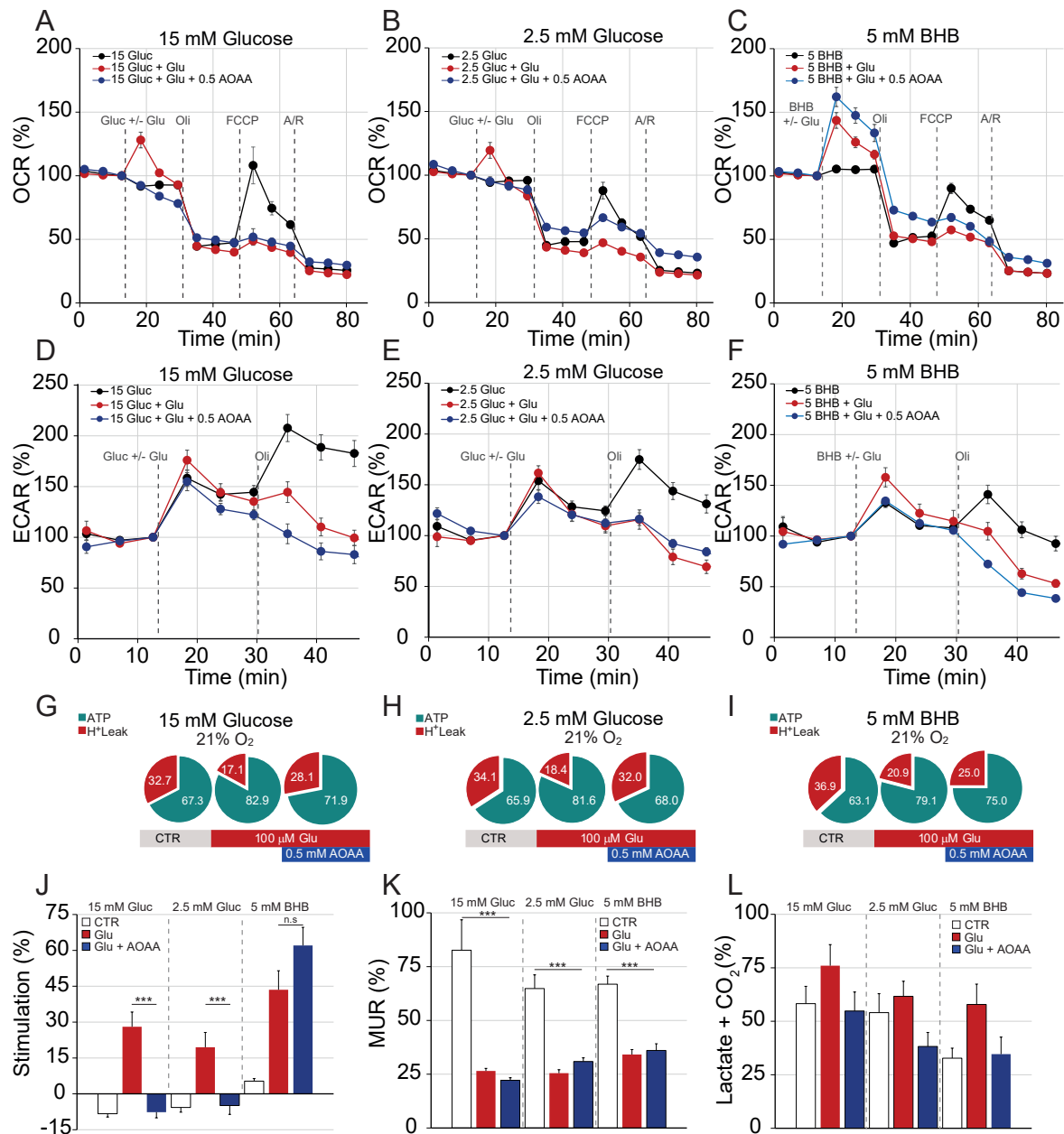

Supplementary figure 1.

2 columns (17.6 cm)

Supplement: Supplementary file 2 — Supplementary Figure S1. [file 41598_2023_46776_MOESM2_ESM.pdf]
